# Occipital lobe epilepsy secondary to Posterior Reversible Encephalopathy Syndrome (PRES) during a post-partum eclampsia in Mali (West Africa)

**DOI:** 10.1186/1756-0500-6-321

**Published:** 2013-08-13

**Authors:** Maïga Youssoufa, Kuate Tegueu Callixte, Napon Christian

**Affiliations:** 1 Neurology Department, CHU Gabriel Touré, Bamako, Mali and Faculty of Medicine pharmacy and Odonto-stomatology, University of Bamako, Bamako, Mali; 2 Neurology Department, Douala Laquintinie Hospital and Faculty of Medicine and Biomedical Sciences, University of Yaoundé I, Yaoundé, Cameroon; 3 Neurology Department, CHU Yalgado Ouédraogo, Ouagadougou, Burkina Faso and Faculty of Medicine, Ouagadougou, Burkina Faso

**Keywords:** Eclampsia, PRES, Occipital lobe epilepsy, Mali, Sub Saharan Africa

## Abstract

**Background:**

Eclampsia is known to cause posterior reversible encephalopathy syndrome (PRES) that is often associated with an extensive neurovascular damage affecting preferably posterior regions, often leading to reversible cortical blindness. In spite the magnitude of these lesions, post eclamptic symptomatic epilepsy is rare. We therefore report a case of symptomatic occipital lobe epilepsy secondary to PRES.

**Case presentation:**

A 39-year-old female right handed teacher who presented with headache of progressive onset, phosphenes, rapid decline of visual acuity to blindness, vomiting, repeated generalized tonic-clonic seizures followed by altered consciousness and very high blood pressure (HBP) of 240/120 mmHg, all of which started about 12 hours following a normal delivery. Nine months later, the patient presented with paroxysmal visual symptoms predominating in the right visual field followed by partial tonic clonic seizures with secondary generalization and recurrence of partial occipital lobe seizures. The pathophysiologic mechanism of irreversible tissue damage during PRES syndrome could result from a combination of events including the delay for early treatment, inadequate antihypertensive drugs that could worsen the brain damage by hypo perfusion, inadequate or delayed treatment for seizures or status epilepticus.

**Conclusion:**

Despite its high incidence in the third world, eclampsia is not a usual cause of epilepsy. Our case is the first description of post eclamptic occipital lobe epilepsy in Africa. With this report, we draw practitioners’ attention on this rare complication.

## Background

Eclampsia is one of the complications of high blood pressure associated with pregnancy. It is an acute paroxystic pathology characterized by seizures and altered mental status and even coma. It remains a common disease in sub-Saharan Africa, with an incidence estimated at 8 per 1000 new births with significant maternal and perinatal complications due to shortcomings in the quality of care offered to the women with this ailment [1,2]. However, in developed countries, the incidence of eclampsia is between 0.2 and 0.5 per 1000 new births [3-5] and tends to decrease due to optimized prenatal care [2,4]. In eclampsia, neurological involvement is the most dreaded and may consist of a posterior reversible encephalopathy syndrome (PRES) whose reversibility is not as constant as in the previously described by Hinchey and collaborators in 1996 [6].

PRES is characterized clinically by the presence of headache, seizures, loss of consciousness, cortical blindness in a woman with high blood pressure in the third trimester of pregnancy, and radiological findings in such patients are predominantly posterior cerebral edema all of which are reversible within three months [7].

This clinical and radiological entity though rare is becoming more and more described with the contribution of brain magnetic resonance imaging (MRI) or cerebral computerized tomographic (CT) scan. Given the extensive brain involvement in PRES and the fact that it has been described to be reversible, there however can occur some unusual situations where there is irreversible damage to the brain, mostly related to inappropriate management at the acute phase [7]. As such, cases of symptomatic post-eclamptic or post-PRES epilepsy have been reported most of which are temporal lobe epilepsies [8,9] which is paradoxical because in PRES, though the lesions are found in the posterior cerebral vascular topography during the acute phase of the disease, symptomatic occipital lobe epilepsy are rare [8].

We therefore report a case of symptomatic occipital lobe epilepsy secondary to PRES.

### Case presentation

**Figure 1:**
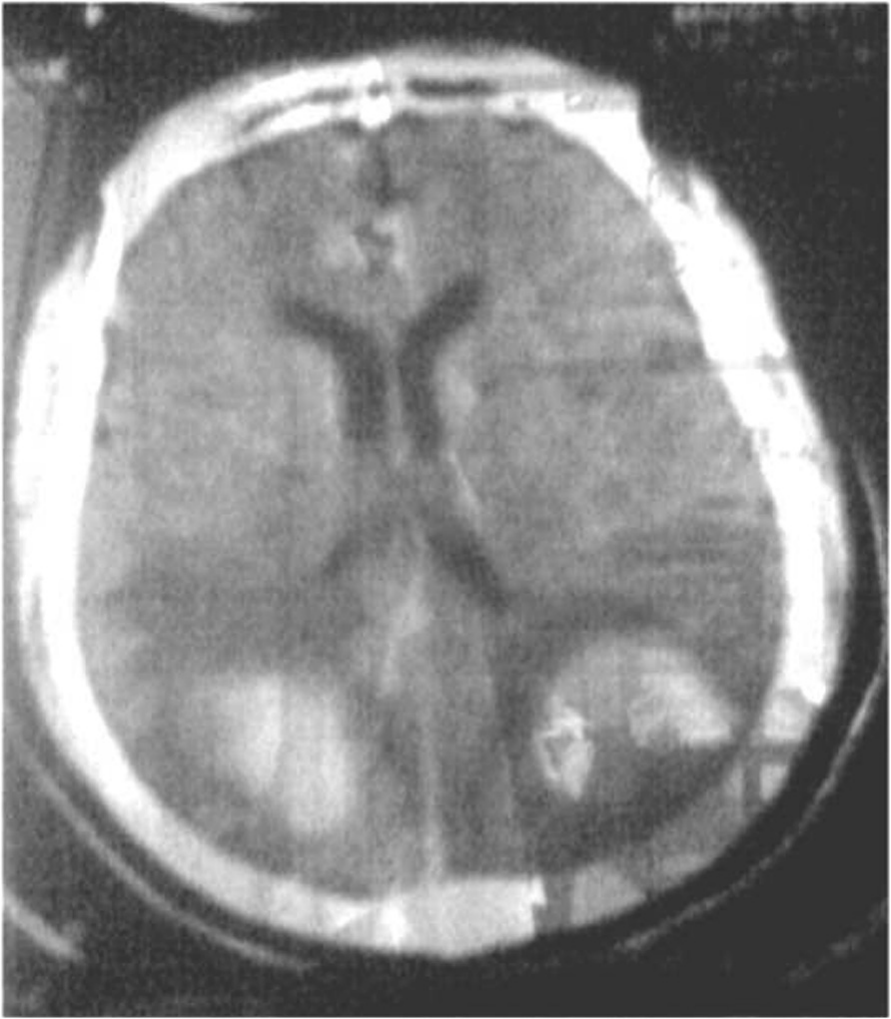
**Brain Computerized tomography showing two bilateral hematoma of parieto**-**occipital region with significant peripheral oedema.**

A 39-year-old female right handed teacher who presented with headache of progressive onset, phosphenes, rapid decline of visual acuity to blindness, vomiting, repeated generalized tonic-clonic seizures followed by altered consciousness and very high blood pressure (HBP) of 240/120, all of which started about 12 hours following a normal delivery. In her past obstetrical history, she is a mother of 7 living children with unremarkable events during her pregnancies except for similar severe headaches that occurred a few hours following her last delivery of twins in June 2010. Concerning her recent gestation, it is worth noting that follow up was irregularly and at the third trimester, she was diagnosed of moderate proteinuria and HBP. Hypertensive encephalopathy was presumed. A computerized tomographic (CT) scan of the brain was immediately requested which showed bilateral symmetric occipital hematoma with significant peripheral oedema (Figure 1). Chest X-ray and electrocardiogram showed no abnormality. Laboratory work-up among which complete blood count (CBC), prothrombin time (PT), liver enzymes, blood urea and creatinine, blood glucose level, serum electrolytes, Thyroid Stimulating Hormone were unremarkable. HIV 1&2 serology as well as malaria diagnostic tests were negative. Her 24 hours proteinuria was 0.4 g. She was admitted into the intensive care unit (ICU) and initial treatment comprised IV Nicardipine and magnesium sulfate. Evolution on this treatment was marked by a fall in Blood Pressure within 6 hours to 130/90 mmHg, subsidence of seizures and she was discharged from the ICU to the ward after 72 hours though on there was persistence of blindness, mental confusion and tetra-pyramidal syndrome.

The evolution was favorable with disappearance of headache, seizures, regain of visual acuity and mental status and she was discharge home 15 days after admission. No control CT scan was done due to financial constraints.

Nine months later, she presented on the night, flashing lights, colored and mobile spotlights, more frequent on the right side of her visual field, followed a few minutes later by a brief loss of consciousness and a secondary generalization with 3 episodes of tonic clonic seizures in one night. Ophthalmologic examination was reportedly normal. Due to the recurrence of partial occipital lobe seizures with secondary generalization few days later with the frequency of 2 to 3 seizures per week she was transferred to the neurology unit of the hospital.

**Figure 2:**
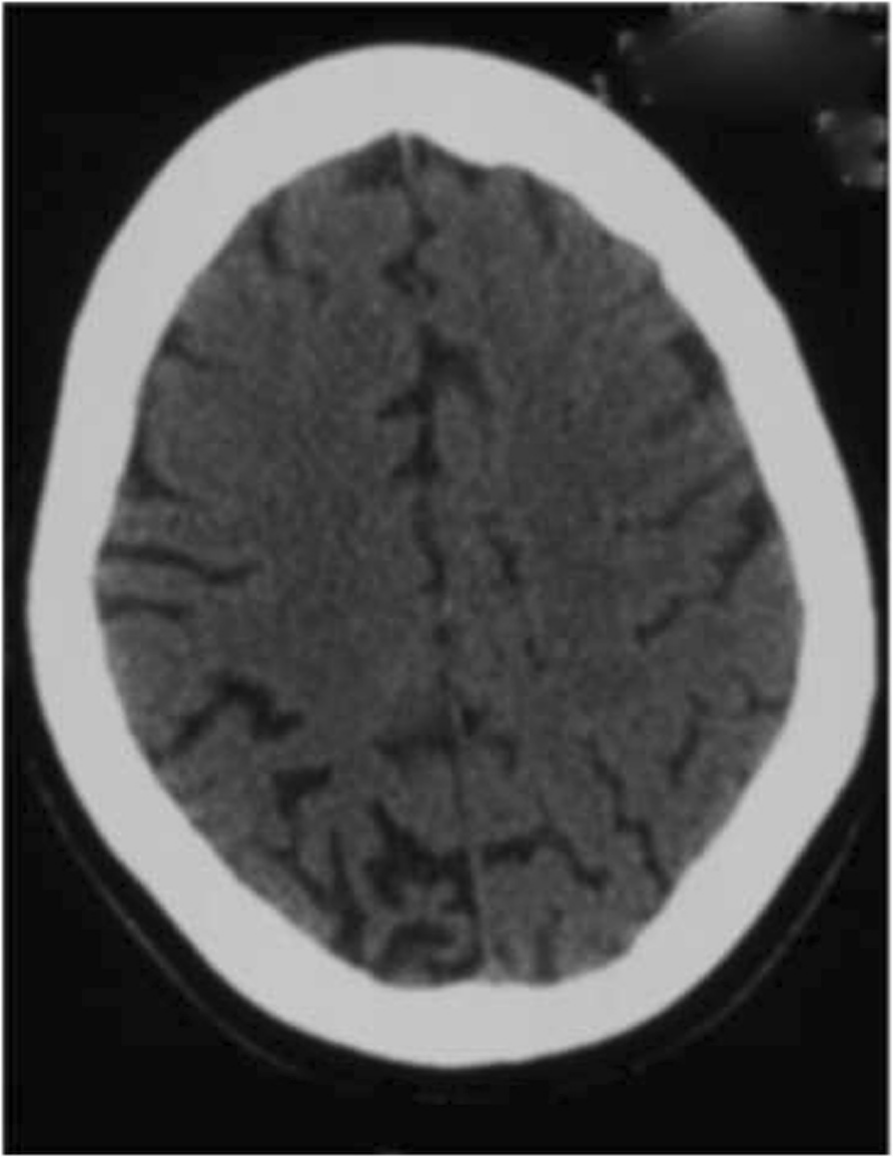
**Brain CT-scan (March 2011): cerebral atrophy of the right posterior region.**

On examination her blood pressure was 120/80 mm Hg with regular pulse at 65 beats per minute and a respiratory rate of 22 regular cycles per minute. Heart, pulmonary and abdominal examination showed no clinical abnormality and neurological examination was unremarkable. Lumbar puncture for cerebrospinal fluid analysis (CSF) was done and it showed no abnormality. Proteinuria and other biological tests like complete blood count, prothrombin time, liver enzymes, blood urea and creatinine, blood glucose level, serum electrolytes were normal. A CT scan was done (about 10 months after the first one) and it showed total regression of hematoma but marked bilateral posterior cortical atrophy predominantly on the right (Figure 2). An electroencephalogram (EEG) was performed immediately after a seizure and it showed generalized slow waves and spikes and a diagnosis of epilepsy secondary to PRES was made and she was placed on Carbamazepine 200 mg twice a day and after six months of treatment, the patient became seizure free and was able to resume her work.

## Discussion

The rationale behind our reporting this case lies in the sequence and unusual nature of events whereby clinically, at the beginning of Posterior Reversible Encephalopathy Syndrome (PRES) there is cortical blindness, radiological finding of bilateral occipital hematoma at the acute phase then its evolution to cortical atrophy in the chronic phase and subsequently the occurrence of late symptomatic occipital lobe epilepsy secondary to vascular sequelae.

Despite the high incidence of eclampsia in Africa [2], we did not find in the literature any reference on symptomatic occipital lobe epilepsy secondary to this disease which is well described in our context.

In this case, evidence for a PRES were: postpartum eclampsia with encephalopathy and seizures, occipital localization of the lesions on CT-scan and reversibility of the disorders [6,10].

Cortical blindness presented by our patient has been reported in the literature. Cunningham and collaborators have reported 15 women with a reversible cortical blindness related to eclampsia during 14 years [11], and in the series by Hinchey and collaborators, out of 15 women, 3 had eclampsia, and 9 patients had visual disturbances [6]. It is unusual that this blindness has an excellent prognosis, with full recovery of visual acuity. From a pathophysiology perspective, the blindness is linked to vasogenic edema and not vasospasm and so its reversibility is linked to the resolution of edema [12].

The occipital lobe epilepsy secondary to eclampsia that we found is exceptional. Till date, two patients with occipital lobe epilepsy post eclampsia have been reported [8] but contrary to our observation, initial brain CT imaging was normal in these two patients as reported by Plazzi and collaborators [8].

The pathophysiologic mechanism of irreversible tissue damage during PRES syndrome is not clear in the literature. The irreversible damage that we found in our patient could result from a combination of events including: delay in early treatment, inadequate anti-hypertensive drugs that could worsen the brain damage by hypoperfusion, inadequate or delayed treatment for seizures or status epilepticus [7,8,13,14].

The anticonvulsant treatment of choice for the management of PRES secondary to eclampsia, is magnesium sulfate. Indeed, a multicenter trial [15], including 1680 eclamptic patients showed clearly the superiority of magnesium sulfate compared to diazepam and phenytoin in the prevention of seizures. In addition, its anti-edematous effect, higher than that of mannitol during PRES is known [16]. Also, a rapid decrease in serum magnesium seems to contemporarily trigger PRES. In the absence of controlled trial, treatment with magnesium sulfate is still elective and in the course of this treatment serum monitoring should be systematically done in cases of PRES. However, the effectiveness of magnesium sulfate in preventing eclampsia in patients with preeclampsia remains controversial [17].

The use of magnesium sulfate, however, raises the problem of the choice of antihypertensive drugs. Their association with injectable calcium channel blockers, generally accepted as the treatment of choice for the management of high blood pressure at the acute phase of stroke [18], involves a risk of aggravation: the effect of calcium channel blockers may be potentiated by magnesium sulfate, then causing a drop in blood pressure with an increase risk of acute brain damage thus, a risk of irreversible sequelae [8,17].

In this case report, an analysis of different stages of the management shows a significant drop in blood pressure (BP) during the acute phase. Indeed, the association of maximum doses of magnesium sulfate and nicardipine resulted in a sharp fall in BP to less than 50% the original figures in less than 6 hours. This potentially damaging attitude is known to aggravate brain lesions, with onset of irreversible damage which potentially could be epileptogenic [7,8,17].

According to his initial description, PRES classically causes reversible damage. However, patient care is not accurate (delayed diagnosis, inappropriate management of BP and or seizures), irreversible sequelae may alter the course of the disease.

The relationship between hypertensive encephalopathy, PRES and symptomatic epilepsy must be confirmed by longitudinal studies [7].

## Conclusion

Eclampsia is still common in developing countries, despite improvement in the management of pregnancy in many countries. This condition may be responsible for PRES, inducing neurovascular lesions typically reversible but delayed diagnosis and/or inadequate management at the initial phase can induce irreversible neurological sequelae which could be potentially epileptogenic. Therefore clinicians’ awareness to possible occurrence of symptomatic occipital lobe epilepsy as sequelae is important. The early diagnosis of PRES as well as optimal management of its acute phase is sine qua non to its reversibility.

### Inform consent

A written informed consent was obtained from the patient for publication of this case report and any accompanying images. A copy of the written consent is available for review by the Editor of this journal.

## Competing interests

The authors declared that they have no competing interests.

## Authors’ contributions

MY conceived of the study, and participated in its design and coordination and helped to draft the manuscript. KTC and NC have made substantial contributions to conception and design; have been involved in drafting the manuscript. All authors read and approved the final manuscript.
